# Enhancing Heat Tolerance of the Little Dogwood *Cornus canadensis* L. f. with Introduction of a Superoxide Reductase Gene from the Hyperthermophilic Archaeon *Pyrococcus furiosus*

**DOI:** 10.3389/fpls.2016.00026

**Published:** 2016-01-29

**Authors:** Xing-Min Geng, Xiang Liu, Mikyoung Ji, William A. Hoffmann, Amy Grunden, Qiu-Yun J. Xiang

**Affiliations:** ^1^Department of Plant and Microbial Biology, North Carolina State University, RaleighNC, USA; ^2^College of Landscape Architecture, Nanjing Forestry UniversityNanjing, China

**Keywords:** antioxidant enzyme, *Cornus canadensis*, genetic transformation, heat tolerance, *Pyrococcus furiosus*, reactive oxygen species (ROS), superoxide reductase (SOR)

## Abstract

Production of reactive oxygen species (ROS) can be accelerated under various biotic and abiotic stresses causing lipid peroxidation, protein degradation, enzyme inactivation, and DNA damage. Superoxide reductase (SOR) is a novel antioxidant enzyme from *Pyrococcus furiosus* and is employed by this anaerobic hyperthermophilic archaeon for efficient detoxification of ROS. In this study, *SOR* was introduced into a flowering plant *Cornus canadensis* to enhance its heat tolerance and reduce heat induced damage. A fusion construct of the *SOR* gene and Green Fluorescent Protein gene (*GFP*) was introduced into *C. canadensis* using *Agrobacterium*-mediated transformation. Heat tolerance of the GFP-SOR expressing transgenic plants was investigated by observing morphological symptoms of heat injury and by examining changes in photosynthesis, malondialdehyde (MDA), and proline levels in the plants. Our results indicate that the expression of the *P. furiosus SOR* gene in the transgenic plants alleviated lipid peroxidation of cell membranes and photoinhibition of PS II, and decreased the accumulation of proline at 40°C. After a series of exposures to increasing temperatures, the *SOR* transgenic plants remained healthy and green whereas most of the non-transgenic plants dried up and were unable to recover. While it had previously been reported that expression of *SOR* in *Arabidopsis* enhanced heat tolerance, this is the first report of the successful demonstration of improved heat tolerance in a non-model plant resulting from the introduction of *P. furiosus* SOR. The study demonstrates the potential of *SOR* for crop improvement and that inherent limitations of plant heat tolerance can be ameliorated with *P. furiosus* SOR.

## Introduction

Reactive oxygen species (ROS), such as superoxide (O_2_^-^), hydrogen peroxide (H_2_O_2_), hydroxyl radical (⋅OH) and singlet oxygen (^1^O_2_), can be generated in the process of aerobic metabolism. ROS production can be accelerated by various environmental stresses, such as drought, heat, high light or a combination of different environmental stresses (Larkindale and Knight, 2002; Carvalho, 2008; Suzuki et al., 2012). Excessive ROS causes oxidative stress, leading to lipid peroxidation, protein degradation, enzyme inactivation, and DNA damage (Apel and Hirt, 2004; Kai et al., 2012). Although ROS does not result in photodamage directly, it inhibits the repair of photodamaged PS II by suppressing the synthesis of PS II proteins in chloroplasts (Murata et al., 2007; Takahashi and Murata, 2008).

On the other hand, ROS can also serve as signaling molecules to control and regulate important biological processes, such as growth, development, and biotic and abiotic stress responses (Baxter et al., 2014). For example, H_2_O_2_ has been implicated in heat-shock-induced cross adaptation to heat, chilling, drought, and salt stress in maize seedlings (Gong et al., 2001). The moderate accumulation of ROS plays an important role in signaling to stress-related genes (Farmer and Davoine, 2007). The conflict between ROS toxicity and signaling roles has led to a tightly regulated equilibrium between ROS production and scavenging. ROS scavenging systems include both enzymatic antioxidants [multiple superoxide dismutases (SOD), ascorbate peroxidase (APX), catalase (CAT), peroxidase (POX)) and other non-enzymatic antioxidants (ascorbate (AsA), glutathione (GSH)].

As the primary product of oxygen reduction, O_2_^-^ is enzymatically disproportionated to H_2_O_2_ and O_2_ by SOD, and this SOD-catalyzed reaction provides the initial defense against ROS in plant cells. Adding SOD to culture medium enhanced the heat tolerance of tobacco cells grown in suspension culture (Vacca et al., 2004). Overexpression of Cu/Zn SOD and APX induced thermotolerance to 42°C heat exposure in transgenic potato plants (Kim et al., 2010). Overexpression of SOD and glutathione reductase (GR), were reported to result in an increased resistance to drought, ozone, low temperature, and high light stress (Van Camp et al., 1996; McKersie et al., 2000; Panchuk et al., 2002). These experiments indicated that modification of ROS scavenging systems can lead to significant changes in oxidative stress tolerance and provide some indication that these approaches can be used to improve plant performance (Allen, 1995).

The anaerobic hyperthermophilic archaeon, *Pyrococcus furiosus*, possesses a system for the detoxification of ROS that is different from the classical defense mechanisms present in aerobes, such as SOD. *P. furiosus* employs a novel enzyme system centered on the enzyme superoxide reductase (SOR) to reduce superoxide molecules to hydrogen peroxide without producing oxygen (Jenney et al., 1999; Im et al., 2009). In contrast, SOD in aerobic organisms produces additional oxygen molecules when it disproportionates the superoxide molecules, which can lead to further generation of ROS. Interestingly, *P. furiosus* SOR, unlike many *P. furiosus* enzymes, was shown to function at low temperature (<25°C; Jenney et al., 1999; Grunden et al., 2005).

Given these apparent advantages that SOR has over SOD, there was interest in determining whether these benefits could provide improved ROS detoxification in plants if *P. furiosus SOR* were functionally expressed in plant tissues. There have been a few studies demonstrating that genes from an archaeal source can be successfully expressed in plant systems (Im et al., 2005; Im et al., 2009). The *P. furiosus SOR* gene was expressed in tobacco cell NT1 culture, which produced a functional protein that retains thermal stability characteristic of the native enzyme. Furthermore, the recombinant GFP-SOR protein was distributed throughout the cytosol and nucleus of the plant cells, and enhanced the survival of the transgenic plant cells to short-term, high temperature exposure and drought stress in *Arabidopsis*.

*Cornus canadensis* is an herbaceous perennial native to northeastern Asia, northern USA, Canada, and Greenland (Kindersley, 2008). In the USA, the *C. canadensis* growth range is extended south to the Rocky Mountains in Colorado and New Mexico, to the mountains of the southern Appalachians in Western Virginia. It thrives in coniferous forests and forest edges with moist, well-drained soils. *C. canadensis* is a common ornamental plant used in the northern USA in hanging baskets or as garden ground cover. However, the species cannot survive in southern regions of the USA (e.g., south of USDA Plant Hardiness Zone 6) and China because of heat stress. In this study, a *GFP-SOR* gene fusion was introduced into *C. canadensis* to determine if SOR could provide improved ROS detoxification and heat tolerance in *C. canadensis*. Successful introduction of *P. furiosus SOR* into this rhizomatous perennial species and observation of improved ROS detoxification and heat tolerance in the transgenic *C. canadensis* provides evidence that genes from an archaeal source can be functionally expressed in diverse plants and that *SOR* can be a beneficial gene in agriculture and horticulture for creation of improved cultivars.

## Materials and Methods

### Generation and Selection of *GFP-SOR* Transgenic Plants

The gene encoding *P. furiosus* SOR (accession no. AE010234) was cloned as a fusion with the green fluorescent protein (GFP) from *Aequorea victoria* into pK7WGF2 as described previously (Im et al., 2005). The *GFP-SOR* expression plasmid harboring a Kanamycin resistance gene in the T-DNA region of the plasmid (provided by Dr. W. Boss’ lab at NCSU) was transformed into *Agrobacterium tumefaciens* EHA105 using the freeze-thaw method (Chen et al., 1994). *GFP-SOR* driven by the 35S promoter was then introduced into *C. canadensis* by *Agrobacterium*-mediated transformation. Explants were excised from young leaves of plants regenerated from tissue culture of materials originally collected from Spruce Knob, Virginia (Feng et al., 2009; Liu et al., 2013). The preparation of explants and the generation of *GFP-SOR* transgenic plants followed our previous protocol with a modified selection (Liu et al., 2013). In this study, 200 μg/ml of kanamycin was used instead of 14 μg/ml of hygromycin in the selection medium. The selected shoots were cultured in rooting medium containing 0.1 μg/ml of IBA. Rooted plantlets were transplanted in soil as previously described (Liu et al., 2013).

### Detection of the *GFP-SOR* Gene in Transgenic *C. canadensis* Plants

Young leaves from each putative transgenic plant were collected for DNA and RNA extraction. DNA was extracted using the DNeasy plant mini kit (Qiagen, Valencia, CA, USA). Genomic DNA PCR was performed using primers GFP-F (TGACCCTGAAGTTCATCTGCACCA) from the *GFP* region and SORe-R (CCACCCTTTCACTCTAAAGTGACTT) from the *SOR* region to amplify the *GFP-SOR* fragment. The PCR products were sequenced to confirm their identity as *GFP-SOR* (ETON Bioscience, Inc.; Durham, NC, USA). RNA was isolated from leaves of transgenic plants using a modified CTAB RNA isolation method (Chang et al., 1993). The products were treated with DNase I (New England Biolab, Beverly, MA, USA) to remove any potential contaminating genomic DNA. First strand cDNA was synthesized using a SuperScript III first-strand synthesis kit (Life Technologies, Carlsbad, CA, USA). RT-PCR was conducted using *SOR* specific primers SOR-F (AAGCACGTCCCCGTTATAGA) and SOR-R (TTTGGGCCGTTTACAGACTC) to detect *SOR* expression. Glyceraldehyde 3-phosphate dehydrogenase gene (*GAPDH*) was used as internal control. Presence of GFP fluorescence in leaves of transgenic plants was verified using a confocal microscope (LSM710, ZEISS, Germany). A leaf sample (1 mm × 1 mm) was placed in deionized water on a microscope slide and imaged using a C-Apochromat 40 × 1.1 NA water immersion objective lens. The samples were excited at 488 nm (argon laser) and emission was collided between 493 and 551 nm.

### Protein Isolation and Immunoblotting

Young leaves from approximately 10-month-old *GFP-SOR*-transgenic and non-transgenic *C. canadensis* plants (∼150 mg each) were harvested and ground using liquid nitrogen with a mortar and pestle. Proteins were extracted from ground powder using a variation of the methanol/acetone extraction procedure as described in Sigma Technical Bulletin (Catalog # PE0230). In lieu of Reagent Type 4, the protein pellet was dissolved in 2X SDS sample buffer with β-mercaptoethanol and boiled for 10 min. The proteins were separated on 12.5% (w/v) polyacrylamide gels and transferred to a PVDF membrane. The membrane was blocked with 5% (w/v) milk in Tris-buffered saline (TBST). Antibodies raised in rabbits against *P. furiosus* SOR (1:10,000 dilution; Cocalico Biologicals, Inc., Reamstown, PA, USA) were used as the primary antibodies, and horseradish POX-conjugated goat anti-rabbit antibody (Thermo Scientific) was used as the secondary antibody at a dilution of 1:15,000. The blot was visualized by exposure to X-ray films after incubating in Clarity Western ECL Substrate (BioRad, Hercules, CA, USA).

### Heat Stress and Thermotolerance Assays

Non-transgenic and transgenic plants were grown at 22°C. Five healthy wild type and SOR transgenic plants of uniform size were chosen and transferred to an artificial climate incubator. The potted plants were placed in a tray with water and exposed sequentially to 30, 35, 37, and 40°C for 24 h at each temperature, with the potting soil maintained constantly moist. Morphological symptoms of heat injury, such as leaf color were observed and recorded photographically every day. Other physiological properties related to plant function and growth were measured after each temperature treatment (see below). After the heat stress experiments, the plants were returned to 22°C. The plants were continuously monitored and observed for 1 week after heat stress. This experiment was conducted twice to confirm the results.

### Measurement of Photosynthesis and Malondialdehyde (MDA) and Proline Levels

To further identify and evaluate heat resistance of *SOR* transgenic *C. canadensis* plants, we measured changes in photoinhibition, membrane lipid peroxidation, and proline content in response to heat stress. As described above, five transgenic plants in individual pots were sequentially exposed to temperatures of 30, 35, 37, and 40°C for 24 h, respectively. As a control, five wild type plants were treated in the same chamber. Leaves from the five individual wild type and SOR transgenic plants were collected after every temperature treatment and were stored in a -80°C freezer for proline and MDA quantitation. This experiment was conducted twice to confirm the results.

To determine the proline content of the dogwood leaves, 0.3 g of the heat-stressed wild type and SOR transgenic leaves were homogenized in 10 ml 3% sulfosalicylic acid. The homogenates were boiled for 15 min and then cooled at room temperature for 30 min. The cooled supernatants were treated with 4 ml of a 2.5% ninhydrin solution dissolved in acetic acid and boiled for 30 min before stopping the reaction by placing the samples on ice. The reaction mixtures were extracted with toluene, and their absorbance determined colorimetrically at 520 nm, which measures a stable, red product from the reaction of proline and hydrindantin dehydrate under acidic conditions. Malondialldehyde (MDA) was determined by the thiobarbituric acid (TBA) method as previously described (Gong et al., 2001; Geng et al., 2009). Three replicates were performed for MDA and proline measurements for each experiment. Statistical analyses were carried out for the values of MDA and proline contents using SPSS17.0 software to calculate the means and standard deviation. The Duncan’s multiple range test (DMRT) was applied to test the significance in differences among treatments (*P* > 0.05).

To quantify photoinhibition of photosystem II, we measured the *F*v*:F*m ratio with a PAM2000 Chlorophyll Fluorometer (Heinz Walz GmbH, Effeltrich, Germany). Prior to measurement, plants were moved to a dark room for 1 h in order to measure minimal fluorescence (*F*o) after dark adaptation and maximal fluorescence (*F*_m_) under saturating flash light. Two measurements were taken on marked leaves of each of the five plants of the wild type and SOR transgenic plants, and the means of the 10 measurements were reported. The measurements were taken during the day between 11:00 AM and 12:30 PM. All measurements were performed with the same instrument settings to allow comparison of the mV output of *F*o and *F*_m_ through time.

## Results

### Transgenic *C. canadensis* Plants Express *GFP-SOR*

**FIGURE 1:**
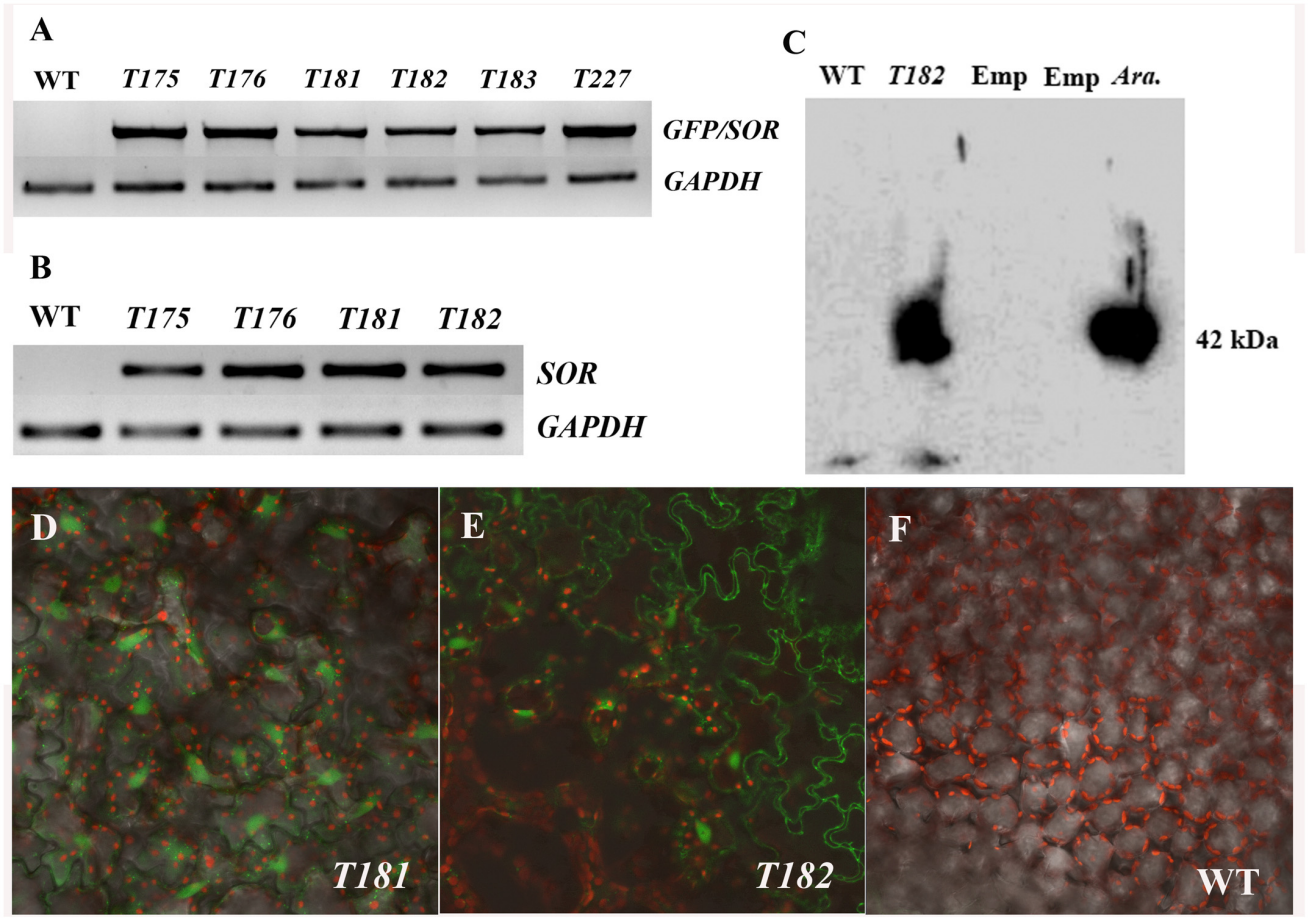
**Verification of transgenic *Cornus canadensis* plants.** **(A)** Green fluorescent protein (*GFP)-*Superoxide reductase (*SOR)* fragments were amplified from transgenic plants using primers eGFP-F from the *GFP* region and SORe-R from the *SOR* region. Transgenic plants *T175*, *T176*, and *T227* were developed from callus 1. *T181*, *T182*, and *T183* were developed from callus 2. WT indicates non-transgenic plants regenerated by tissue culture. *GAPDH* (Glyceraldehyde 3-phosphate dehydrogenase) gene was used as internal control. **(B)** High expression of *SOR* was detected in transgenic plants by RT-PCR using *SOR* specific primers SOR-F and SOR-R. **(C)** Western blot analysis performed using *GFP-SOR* transgenic *Arabidopsis* as positive control. Emp. indicates an empty lane without any sample loaded. *Ara.* Represents *Arabidopsis GFP-SOR* transgenic plants. **(D–F)** GFP Fluorescence was observed in leaves of transgenic plants **(D,E)** using a confocal microscope (LSM710, ZEISS, Germany). No GFP fluorescence was detected from non-transgenic plants **(F)**.

The fusion of *GFP-SOR* cDNA was introduced into *C. canadensis* by *Agrobacterium*-mediated transformation. More than 30 putative transgenic plants from two different calli were obtained and successfully potted in soil. The presence of the *GFP-SOR* genes in the transgenic plants was confirmed in all of the selected transgenic plants by amplifying the *GFP-SOR* fusion using GFP-F and SORe-R primers that are complementary to portions of the *GFP* and the *SOR* gene sequences, respectively. Examples of this PCR confirmation are shown in **Figure 1A** (T175, Y176, and T227 from callus 1; T181, T182, and T183 from callus 2). To demonstrate *SOR* expression in the transgenic *C. Canadensis* plants, RT-PCR was performed using *P. furiosus SOR* gene specific primers. Expression of SOR was detected only in the transgenic *C. canadensis* plants, while no product was observed for the WT *C. canadensis* plants (**Figure 1B**). Confirmation of functional expression of GFP in the *GFP-SOR* transgenic plants was established using confocal microscopy. Fluorescence was observed only in leaf cells of the transgenic plants (**Figures 1D–F**).

Successful *GFP-SOR* transformation in *Arabidopsis* was previously reported, and the functional SOR was detected in transgenic lines (Im et al., 2009). To verify GFP-SOR in the transformed *C. canadensis* plants, Western blot analysis was performed using those same *Arabidopsis* transgenic lines as a positive control. A distinct band was detected for the *C. canadensis* transgenic plant sample, which corresponds to the size observed in the *Arabidopsis GFP-SOR* transgenic line (∼42 kDa, where the GFP protein is 27 and 15 kDa for SOR; **Figure 1C**). The antiserum used recognizes the SOR in *C. canadensis* and the *Arabidopsis SOR* transgenic lines, while the non-transgenic *C. canadensis* sample does not show a detectable band. This result, in addition to the observed *SOR* expression and *GFP* florescence in the putative transgenic plants, demonstrated that the *GFP-SOR* gene fusion was successfully introduced in these *C. canadensis* plants.

### Heat Stress Tolerance in *GFP-SOR* Transgenic *C. canadensis* Plants

**FIGURE 2:**
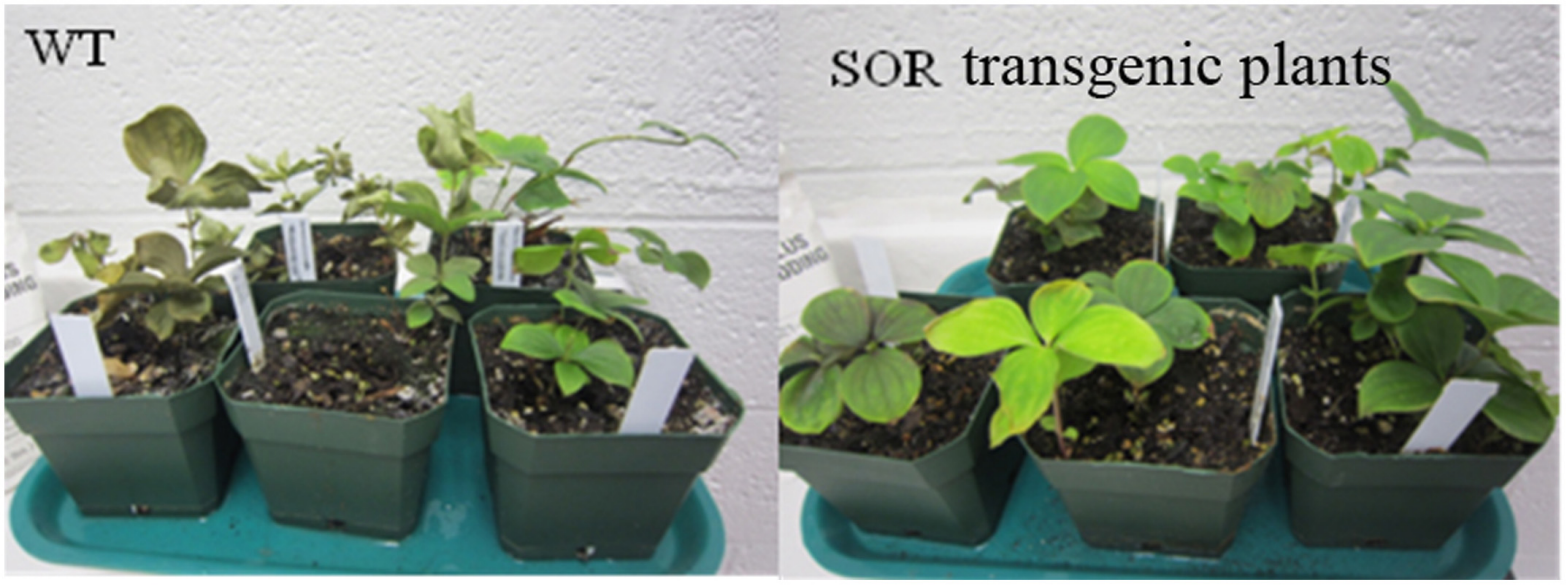
**Heat stress tolerance of SOR transgenic *C. canadensis* plants.** Both transgenic and non-transgenic plants were heat stressed at increasing temperatures of 30, 35, 37, and 40°C. Each temperature was applied for 24 h before being incremented to the next level. Images were taken immediately after 4 days heat stress.

Under non-stressed conditions the growth rate and phenotypic appearance of the wild type control and *GFP-SOR* transgenic plants were indistinguishable. In an effort to determine whether expression of *GFP-SOR* conferred a measure of heat tolerance to *C. canadensis* similar to that observed in *GFP-SOR Arabidopsis* (Im et al., 2009), a heat stress experiment was conducted in which transgenic and non-transgenic plants were exposed to successively increasing temperatures of 30, 35, 37, and 40°C. All plants were exposed to each temperature for 24 h before being incremented to the next level. By the end of this treatment, most WT (non-transgenic) plants withered, while *GFP-SOR* transgenic plants remained green (**Figure 2**). After heat stress, all of the *GFP-SOR* transgenic plants quickly recovered, but fewer than 20% of the non-transgenic plants recovered.

### Changes in Free Proline and MDA Content in Heat-Stressed *C. canadensis*

**FIGURE 3:**
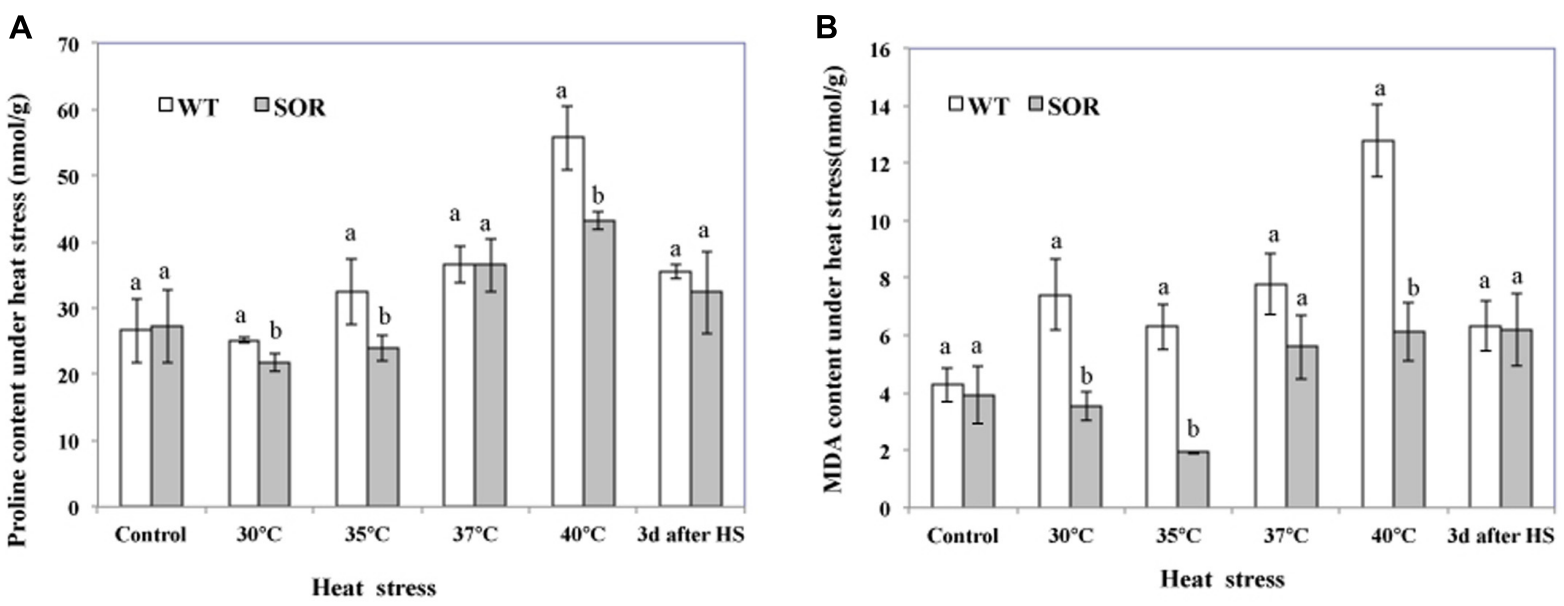
**Malondialdehyde (MDA) and proline levels.** Both transgenic and non-transgenic plants were exposed to temperatures of 30, 35, 37, and 40°C for 24 h, respectively. Leaves from each pot were collected at each temperature treatment for the measurement of proline **(A)** and MDA **(B)**. After heat stress, plants were move to chamber at 22°C for recover. The Duncan’s multiple range test was applied to compare significant differences between WT and GFP-SOR expressing *C. canadensis* plants. Letters a and b indicate significant difference at the *p* < 5% level. The Duncan’s multiple range test was applied to determine the significance of differences between WT and GFP-SOR expressing plants. Letters a and b indicate significant difference at the *p* < 5% level between the levels in WT and transgenic plants at a given temperature. If only a is marked for the levels in WT and transgenic plants at a given temperature, the difference is not significant.

Proline, an effective osmotic adjustment compound, is known to be produced in a variety of higher plants and to accumulate to high concentrations in response to environmental stresses (Kishor et al., 2005). In this study, we evaluated the proline levels in the non-transgenic and transgenic *C. canadensis* plants to determine whether the expression of *GFP-SOR* had a protective effect in the heat-stressed plants as reflected by lower proline levels. Our results indicated that the proline levels in transgenic plants were all lower than non-transgenic plants, except at 22°C (control) and 37°C (**Figure 3A**). In addition, the proline level did not increase in transgenic plants until the plants were exposed to 37°C temperatures, while it began to increase upon exposure to 35°C in the non-transgenic plants. Upon heat exposure at 40°C for 24 h, the plants were returned to the chamber maintained at 22°C, and the proline content was measured again 3 days later. In this case, proline levels in both the *GFP-SOR* transgenic and control plants were somewhat lower than the treatment at 40°C, but proline levels in the control plants remained higher than the levels observed in the unstressed plants.

The MDA content of plant cells is considered to be an indicator of oxidative damage (Moller et al., 2007). More specifically, MDA can be used as a suitable marker for membrane lipid peroxidation. Our MDA analyses indicated that the MDA levels in the *GFP-SOR* plants were much lower than the non-transgenic plants (WT) at all temperature points of the heat stress (from 30 to 40°C; **Figure 3B**). After 3 days of recovery from the heat stress, the MDA levels in WT plants decreased to a similar level as observed in the transgenic plants, but they were still slightly higher than the level seen prior to the heat stress (22°C). This pattern of changing levels of the MDA is consistent with the observation of heat stress tolerance of the *GFP-SOR* expressing plants (**Figure 2**).

### Effect of Heat Stress on Photosynthesis in WT and *GFP-SOR* Expressing *C. canadensis* Plants

Chlorophyll fluorescence (*F*v*/F*m), and the base fluorescence (*F*o) are physiological parameters that have been shown to correlate with heat tolerance (Yamada et al., 1996). At our control temperature (22°C), the base fluorescence (*F*o) in the leaves of the GFP-SOR expressing plants was slightly higher than those of WT plants. The *F*o of the transgenic plants began to increase after exposure to heat stress at 35°C, while the *F*o of the WT plants began to increase at a 30°C temperature exposure (**Table 1**). The *F*o of the WT plants was much higher than transgenic plants after exposure to the 40°C of heat stress. Chlorophyll fluorescence, the ratio of variable fluorescence to maximum fluorescence (*F*v*/F*m), decreased as the stress temperature increased, and this decrease was more pronounced in WT plants compared to the *GFP-SOR* expressing plants.

**Table 1:** Effects of heat stress on photoinhibition of photosynthesis as indicated by chlorophyll fluorescence.

		Control	30°C	35°C	37°C	40°C
*F*o (mV)	WT	271.4	302.3	343.9	345.7	384.4
	SOR	310.8	301.2	353.9	334.7	355.2
*F*m (mV)	WT	1367.5	1480.0	1239.8	937.5	612.9
	SOR	1530.3	1429.4	1484.7	1200.3	972.0
*F*v*/*m	WT	0.802	0.795	0.720	0.600	0.335
	SOR	0.805	0.788	0.756	0.704	0.590


## Discussion

Production of ROS constitutes a major plant response to heat stress. Generation of ROS is symptomatic of cellular injury due to heat stress (Liu and Huang, 2000). In *Phaseolus vulgaris*, ROS content increased upon exposure to heat stress temperatures of 46–48°C, which further led to lipid peroxidation in membranes and accumulation of MDA (Nagesh Babu and Devaraj, 2008; Kumar et al., 2011). These ROS are continuously reduced/scavenged by plant antioxidative defense systems which maintain them at certain steady-state levels under stressful conditions (Mittler et al., 2004). Protective roles of antioxidant enzymes in response to temperature stress have been reported for a number of plants (Larkindale and Knight, 2002; Almeselmani et al., 2006; Camejo et al., 2006). According to Kaushal et al. (2011), oxidative damage, measured as lipid peroxidation and hydrogen peroxide concentration, increased, and this is coupled with inhibition of antioxidant enzyme activities and decreased levels of non-enzymatic antioxidants. Supplementation with ascorbic acid mitigated oxidative stress and increased heat tolerance (Kumar et al., 2011). Overexpression of Cu/Zn SOD and APX induced thermotolerance in transgenic potato plants (Kim et al., 2010); however, a decrease in the activities of enzymatic antioxidants (SOD, CAT, APX, GR) was observed as a result of their denaturation at higher temperature (Kaushal et al., 2011). These reported findings suggest that the functional temperature range and production of plant enzymes to remove O_2_^-^ are limited by endogenous mechanisms regulating either enzyme function or gene expression (Im et al., 2009).

To circumvent the inherent limitations of endogenous antioxidant processes, we sought to enhance plant tolerance with *P. furious* SOR, which has a functional temperature range of 4–100°C (Im et al., 2009). In the present study, the *SOR* gene from *P. furiosus* as a fusion with *GFP* was successfully introduced into *C. canadensis* plants by *Agrobacterium*-mediated transformation. *SOR* expression in transgenic plants was verified by both RT-PCR and Western analyses. The expression of *SOR* alleviated lipid peroxidation measured by MDA and increased the heat tolerance of *C. canadensis* in transgenic plants.

Heat stress increased proline levels in *C. canadensis*, but this accumulation was decreased in plants expressing *GFP-SOR* than in WT. This evidence suggests that SOR may have mitigated the degree of water stress experienced by these plants under high temperatures. However, proline can also act as a potent non-enzymatic antioxidant (Rejeb et al., 2014). Proline synthesis may buffer cellular redox potential under heat and other environmental stresses (Wahid and Close, 2007). Proline exogenously applied to tobacco culture cells resulted in decreased lipid peroxidation but increased SOD and CAT activities (Islam et al., 2009). Supplementation with proline considerably reduced H_2_O_2_ production and showed a decrease in oxidative injury coupled to elevated levels of antioxidants in sugarcane (Rasheed et al., 2011). Although proline accumulation has been well documented to have positive roles in enhancing plant tolerance, according to Lv et al. (2011), proline accumulation under heat stress decreases the thermotolerance of *Arabidopsis* seedlings and is involved in ROS production.

Photosynthesis is known to be one of the most heat-sensitive processes occurring in plants, and it can be completely inhibited by high temperatures before other symptoms of the stress are detected (Berry and Bjorkman, 1980). The photochemical reaction in thylakoid lamellae and carbon metabolism in the stoma of chloroplasts have been suggested as the primary sites of injury at high temperature (Wise et al., 2004). This injury, which is associated with the production of ROS (Camejo et al., 2006; Guo et al., 2006), is typically manifested as a decline in maximum quantum efficiency *(F*v*/F*m). Following the increase of heat stress, *F*v*/F*m decreased gradually, while *F*o increased in the WT and *GFP-SOR* transgenic plants; however, the expression of the *SOR* gene reduced the decline in the *F*v*/F*m ratio in transgenic plants (**Table 1**).

Expression of the *GFP-SOR* fusion promoted heat tolerance not only by alleviating the damage to cell membranes and photoinhibition of *C. canadensis* plants but also by maintaining the steady-state level of ROS, which enables ROS to act as signaling molecules but not to accumulate to a level that gives rise to cytotoxic effects (Mittler et al., 2004). It has been well documented that ROS act as signaling molecules that control and regulate some biological processes, as well as biotic and abiotic stress responses (Baxter et al., 2014). ROS generated in response to abiotic stress act as signals to induce the production of glutamate for proline synthesis in tobacco and grapevines (Skopelitis et al., 2006). ROS may be used as a general signal to prime or activate other signaling networks such as peptides, hormones, lipids, cell wall fragments and others (Mittler et al., 2011). Oxidative stress defense responses might be a central component mediating cross-tolerance (Bowler et al., 1992). Therefore, *GFP-SOR* transgenic *C. canadensis* plants may have an enhanced tolerance to drought, salt, and other stresses, as has been reported in *Arabidopsis* (Im et al., 2009).

## Conclusion

This is the first report of the successful demonstration of improved heat tolerance in a non-model plant that has been transformed with *P. furiosus* SOR. The study supports that the inherent limitations in heat tolerance performance of plants can be moderated with *P. furious* SOR thereby increasing heat tolerance. The study further demonstrates the potential of SOR in crop improvement and provides a promising new cultivar of *C. canadensis* for cultivation in southern regions.

## Author Contributions

X-MG conducted heat stress experiments, collected physiological data on heat tolerance, prepared draft figures, and drafted the first version of the manuscript; Q-YX generated and propogated the transgenic plants, conducted the gene sequencing and expression analyses, finalized figures, and manuscript revision. X-MG and Q-YX contributed equally to the projects. MJ conducted the protein analysis and contributed to manuscript writing. WH supervised the experiments on physiological analyses and contributed to the manuscript writing. AG contributed to project planning, supervised the protein analysis, and manuscript writing and structuring. Q-YX initiated and directed the project, contributed to manuscript writing and finalizing.

## Conflict of Interest Statement

The authors declare that the research was conducted in the absence of any commercial or financial relationships that could be construed as a potential conflict of interest.
