# Corrigendum: Enhancing Heat Tolerance of the Little Dogwood *Cornus canadensis* L. f. with Introduction of a Superoxide Reductase Gene from the Hyperthermophilic Archaeon *Pyrococcus furiosus*

**DOI:** 10.3389/fpls.2016.00226

**Published:** 2016-02-24

**Authors:** Xing-Min Geng, Xiang Liu, Mikyoung Ji, William A. Hoffmann, Amy M. Grunden, Qiu-Yun J. Xiang

**Affiliations:** ^1^Department of Plant and Microbial Biology, North Carolina State UniversityRaleigh, NC, USA; ^2^College of Landscape Architecture, Nanjing Forestry UniversityNanjing, China

**Keywords:** antioxidant enzyme, *Cornus canadensis*, genetic transformation, heat tolerance, *Pyrococcus furiosus*, reactive oxygen species (ROS), superoxide reductase (SOR)

The Author Contributions were erroneously reported in the original article. The statement that reads: “Q-YX generated and propagated the transgenic plants, conducted the gene sequencing and expression analyses, finalized figures, and manuscript revision. X-MG and Q-YX contributed equally to the projects” should have been provided as “XL generated and propagated the transgenic plants, conducted the gene sequencing and expression analyses, figure finalization, and manuscript revision. XG and XL contributed equally to the projects.” XL is a research specialist in Q-YX's lab.

This correction does not affect the scientific validity of the results.

## Author contributions

All authors listed, have made substantial, direct and intellectual contribution to the work, and approved it for publication.

### Conflict of interest statement

The authors declare that the research was conducted in the absence of any commercial or financial relationships that could be construed as a potential conflict of interest.

